# Examining evidence use in vaccine policymaking in Kenya: a qualitative case study

**DOI:** 10.1093/heapol/czag100

**Published:** 2026-08-10

**Authors:** Dennis Waithaka, Simon Lewin, Jacinta Nzinga, Unni Gopinathan, Claire Glenton, Edwine Barasa, Benjamin Tsofa

**Affiliations:** Health Economics Research Unit, KEMRI Wellcome Trust Research Programme, P.O. Box 43640-00100, Nairobi, Kenya; Department of Health Sciences Ålesund, Norwegian University of Science and Technology (NTNU), P.O. Box 1517, 6025 Ålesund, Norway; Centre for Epidemic Interventions Research, Norwegian Institute of Public Health, P.O. Box 222 Skøyen, 0213 Oslo, Norway; Health Systems Research Unit, South African Medical Research Council, P.O. Box 19070, Tygerberg 7505, Cape Town, South Africa; Health Economics Research Unit, KEMRI Wellcome Trust Research Programme, P.O. Box 43640-00100, Nairobi, Kenya; Liverpool School of Tropical Medicine, Pembroke Place, Liverpool L3 5QA, United Kingdom; Department of Health Sciences Ålesund, Norwegian University of Science and Technology (NTNU), P.O. Box 1517, 6025 Ålesund, Norway; Global Health Cluster, Division for Health Services, Norwegian Institute of Public Health, P.O. Box 222 Skøyen, 0213 Oslo, Norway; Department of Health and Functioning, Western Norway University of Applied Sciences, Postbox 7030, 5020 Bergen, Norway; Health Economics Research Unit, KEMRI Wellcome Trust Research Programme, P.O. Box 43640-00100, Nairobi, Kenya; Centre for Tropical Medicine and Global Health, Nuffield Department of Medicine, University of Oxford, Big Data Institute, Old Road Campus, Oxford OX3 7LF, United Kingdom; KEMRI Centre for Geographic Medicine Research, KEMRI Wellcome Trust Research Programme, P.O. Box 230-80108, Kilifi, Kenya

**Keywords:** evidence, policy, vaccine decision-making, civil society, Kenya

## Abstract

Although evidence-informed health policymaking is widely promoted, evidence use in vaccine policymaking and the institutional and actor dynamics shaping it remain poorly understood. This study investigates what types of evidence was used and how it was used in vaccine policymaking in Kenya. Using a qualitative case study design, we focused on the introduction and roll-out of HPV and malaria vaccines. Data collection included 34 in-depth interviews with national Ministry of Health officials, Kenya National Immunization Technical Advisory Group members, County Expanded Programme of Immunization logisticians, international partners, and local civil society organizations; 417 hr of observation of vaccine-related policy and planning meetings; and a review of relevant policy and planning documents. Framework analysis was used to analyse the data, guided by two analytical frameworks: (i) a spectrum approach for categorizing evidence along two dimensions—scientific to tacit and global to local—and (ii) Weiss’s models of research use, which interpret patterns of evidence utilization in policymaking. The findings show that international actors, through their financial and technical authority, substantially influenced national policymakers’ adoption of global guidance and scientific evidence. Local programmatic expertise played a crucial role in validation and contextualization of global guidance and scientific evidence to fit local contexts. The dominance of medical and public health professionals in decision-making spaces led to the prioritization of evidence on vaccines’ public health benefits over socio-political considerations. Local civil society’s influence in vaccine policymaking was limited, with its community-based experiential knowledge primarily informing advocacy, communication, and social mobilization, rather than vaccine prioritization and policy decisions. We conclude that strengthening evidence-informed policymaking requires more inclusive approaches that value diverse evidence types. Addressing power imbalances that shape what counts as credible evidence and integrating local civil society perspectives throughout the policymaking process can enhance the legitimacy, acceptability, and effectiveness of policy decisions.

Key messages

- Despite widespread calls for evidence-informed policymaking, there is limited understanding of how different types of evidence are used in health policy processes in low- and middle-income countries.
- This study found that international actors strongly influenced the use of global guidance documents and scientific evidence in Kenya’s vaccine policymaking, while local tacit knowledge was essential for adapting this evidence to contextual realities. However, civil society’s role and its evidence were often marginalized.
- Strengthening vaccine policymaking requires deliberate efforts to integrate both scientific and tacit evidence, alongside reforms to funding and technical support structures that better value local knowledge and civil society engagement to improve policy acceptance.

## Introduction

Evidence-informed decision-making has become a common aspiration for health policymaking over recent decades (Oxman et al. 2007, 2009, WHO 2007, 2017, 2021). However, few studies of evidence use examine policymaking as it unfolds in real time through observational methods, draw on relevant theoretical frameworks, and consider the full range of evidence, including experiential knowledge (Verboom and Baumann 2022). Vaccine policymaking provides an illustrative case for examining evidence use because its institutional arrangements and actor networks may expose tensions both between global guidance and local realities, and between different forms of evidence, ranging from scientific expertise to experiential knowledge.

Vaccine decision-making structures have historically had a strong technocratic orientation, characterized by the dominance of biomedical and public health expertise (Freed 2008, Bryson et al. 2010, Burchett et al. 2012a, 2012b). This technocratic orientation was institutionalized through global actors such as the World Health Organization (WHO), which from the 1970s guided countries to establish national immunization programmes, disease surveillance systems, regulatory frameworks, and expert advisory bodies (Marti et al. 2024, Waithaka 2025).

The formation of WHO’s Strategic Advisory Group of Experts on Immunization (SAGE) in the late 1990s further positioned expert bodies at the centre of global vaccine policy decisions (Duclos 2010, Marti et al. 2024). SAGE issues global vaccine policy recommendations that shape the priorities of donors (such as Gavi, the Vaccine Alliance), vaccine manufacturers, and governments, particularly in low- and middle-income countries (LMICs) (Marti et al. 2024). Consequently, vaccine introduction timelines in many LMICs tend to follow a predictable pattern: WHO endorsement, followed by Gavi support, and then national introduction (Munira and Fritzen 2007, Burchett et al. 2012b). Supplementary file S1 (see online supplementary material) illustrates this pattern in Kenya, where the sequence of routine vaccine introductions has closely mirrored global trends, reflecting the continued influence of global technocratic guidance and financing on national vaccine policy.

To strengthen national ownership of vaccine policy decisions, WHO later promoted the establishment of National Immunisation Technical Advisory Groups (NITAGs), modelled on SAGE (Duclos 2010, SIVAC 2014). NITAGs were intended to create national mechanisms through which local experts could systematically appraise and contextualize global recommendations for their own settings (Duclos 2010, SIVAC 2014). While NITAGs enhanced the technical rigour of national vaccine policymaking, studies suggest they often privilege biomedical evidence while marginalizing other forms of evidence, including local civil society perspectives that can improve policy relevance and acceptability (Freed 2008, Ward et al. 2019, Panero et al. 2020). This concern is particularly important because adapting global recommendations to national contexts requires attention not only to biomedical evidence but also to local contextual and experiential knowledge (Klugar et al. 2024, Kredo et al. 2024).

At the same time, shifts in the vaccine policy context have revealed the limitations of a globally driven or technocratic approach that does not sufficiently consider local realities and knowledge. As immunization agendas expand to new population groups (adolescents and adults) and vaccine costs rise, decision-making increasingly requires sensitivity to local socio-cultural contexts, health system constraints, and diverse stakeholder perspectives (Ward et al. 2019, Panero et al. 2020, Tiwana and Smith 2024). These emerging challenges underscore the need for a broader evidence base that integrates scientific and experiential insights to ensure that policies are not only technically sound but also locally acceptable, equitable, and sustainable (Gopinathan et al. 2024, Lewin et al. 2024).

Existing studies on vaccine-related policymaking have largely focused on the structure and functioning of immunization advisory bodies (Freed 2008, Smith et al. 2009, Bryson et al. 2010, Muangchana et al. 2010, Panero et al. 2020) and on decision-making criteria for vaccine introduction (Piso and Wild 2009, Bryson et al. 2010, Burchett et al. 2012a, González-Lorenzo et al. 2015, Donadel et al. 2021). However, less attention has been paid to how broader institutional arrangements and actors shape evidence use across multiple stages of vaccine policymaking. This study examines what types of evidence were used and how they were used in vaccine policymaking in Kenya.

## Methods

### Conceptual framework

The study used two primary frameworks. The first was the evidence spectrum approach (Fig. 1) to help map the different types of evidence used in policymaking (Waithaka et al. 2026). The evidence spectrum approach was developed to move beyond binary or hierarchical classifications of evidence and to provide a structured way of mapping the diverse forms of evidence used in health policy and systems decision-making. The approach builds on two recurring distinctions in the literature on evidence use: the distinction between tacit and scientific evidence, and the distinction between local and global evidence. It was developed through four iterative stages: reviewing literature on how evidence is defined and categorized; testing initial tacit–scientific and local–global distinctions using empirical and review-based examples of health policy and systems decision-making; research team deliberations to refine these binary categories into continua; and stakeholder feedback on the clarity, relevance, and applicability of the approach. This approach places evidence on a spectrum ranging from scientific evidence—characterized by systematic and transparent data collection and analysis—to tacit evidence, which refers to knowledge that is not easily accessible in written form, grounded in individual experiences or practices, and often conveyed through personal interactions rather than formal documentation (Fig. 1) (Waithaka et al. 2026). Evidence with intermediate levels of systematization, transparency, or independence from individual judgment are positioned near the midpoint of the spectrum (Waithaka et al. 2026). It also places evidence on a spectrum—ranging from local evidence, which is tied to a specific geography (e.g. disease burden in Kenya), to regional or comparable evidence, which comes from settings with comparable geographic or health systems contexts to the local (or decision-making) setting (e.g. disease burden across East Africa), and finally to global evidence, which has limited connection to a specific geography (e.g. global disease burden) (Waithaka et al. 2026). This approach was suitable for this study because it allowed us to describe the range of evidence used in vaccine policymaking without treating evidence types as fixed, binary, or inherently hierarchical categories.

**Figure 1:**
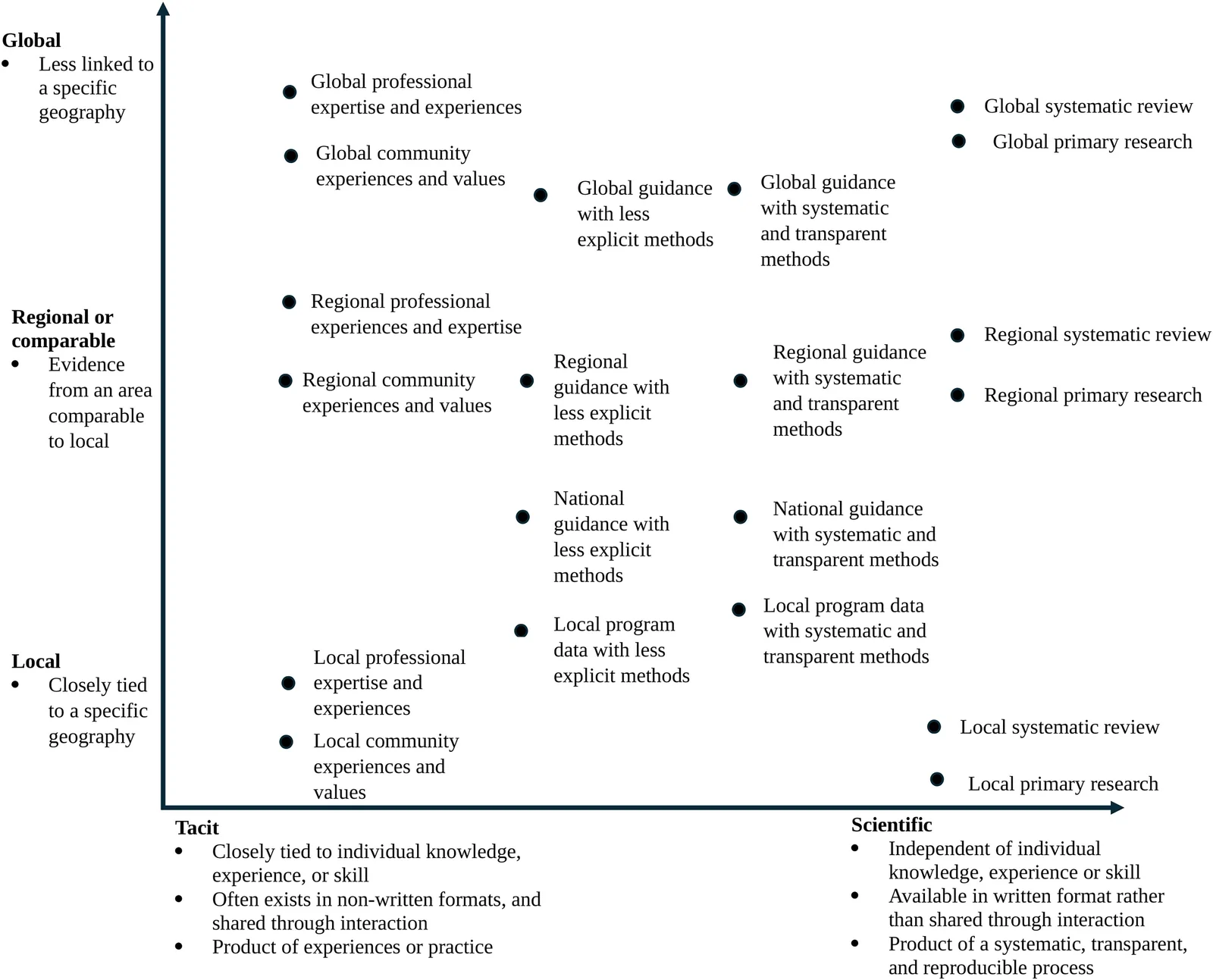
Spectrum approach to evidence categorization with markers for mapping against the *x*-and *y*-axes. Adapted from Waithaka et al. (2026).

Second, we drew on Weiss’s models of research use to capture how evidence and other factors interact to influence vaccine policymaking (Weiss 1979, Weiss et al. 2005). Although the models were originally centred on research evidence, we applied them in this study across both scientific and tacit evidence. We describe below the models that were most relevant to our findings and how we interpreted them; the full set of Weiss evidence-use models is provided in supplementary file S2, see online supplementary material.

Instrumental use refers to the direct application of evidence to inform a decision or action. Interactive use refers to situations where multiple evidence types and actor judgements are considered together through deliberations. Political use occurs when evidence is used to justify, support, or advance a fixed and predefined policy position or pathway. Imposed use refers to situations where an external requirement constrains and influences the evidence, choices, or process.

### Study design

This study employed a qualitative case study design (Yin 2014), focusing on two vaccine policy cases: human papillomavirus (HPV) and malaria vaccines. These were chosen due to differences in their introduction timelines: HPV had been introduced within the past 2 years, while the malaria vaccine was still in the process of introduction. This allowed for both retrospective and prospective elements that enrich the analysis. The two cases also differed in vaccine-specific political and programmatic features that were expected to shape evidence use and decision-making. Malaria vaccination was situated within the more familiar context of childhood immunization and a disease area with longstanding national and donor attention, including support for malaria vaccine development and piloting. In contrast, HPV vaccination represented a life-course immunization intervention targeting adolescent girls, requiring multisectoral delivery arrangements, including school-based strategies, and raising anticipated acceptability concerns linked to vaccination of adolescent girls. These differences provided analytically contrasting cases within the same national immunization programme.

### Study setting

This study was conducted at the National Vaccines and Immunization Programme (NVIP) and the Kenya National Immunisation Technical Advisory Group (KENITAG). NVIP is a division within the Ministry of Health (MOH), and oversees vaccine policy, procurement, and coordination across counties to ensure alignment with national and global immunization goals (MoH 2021). NVIP’s day-to-day operational tasks are organized through four thematic sub-committees: supply chain and logistics; advocacy, communication, and social mobilization (ACSM); monitoring and evaluation; and training and service delivery. NVIP also convenes temporary working groups to spearhead vaccine-specific tasks, such as HPV and malaria vaccine introduction and roll-out processes.

Additionally, NVIP serves as the secretariat for KENITAG (supplementary file S3, see online supplementary material). KENITAG provides recommendations to NVIP on whether to introduce or modify vaccine policy (KENITAG 2015). To make the recommendations, KENITAG uses a structured evidence-to-decision framework (supplementary file S4, see online supplementary material)—a tool for packaging evidence into key domains seen as important to inform decision making (Alonso-Coello et al. 2016).

The NVIP is linked to healthcare facilities through the county and sub-county Expanded Programme of Immunization (EPI) logisticians. The county EPI logistician coordinates vaccine and related supplies (including syringes, safety boxes, cold chain equipment, reporting tools), distribution from national stores to sub-county stores as well as upwards reporting of consumption of these resources (MoH 2021). Similarly, at the sub-county level, the sub-county EPI logistician coordinates the distribution of vaccines and related supplies from the sub-county store to individual facilities as well as upwards reporting of the consumption of these resources (MoH 2021).

### Data collection procedures

Data collection included observations, document review, and interviews that were informed by the study’s conceptual framework. D.W. spent 417 hr conducting observations within NVIP and KENITAG between November 2022 and March 2024 (Table 1).

**Table 1:** Observation events and related vaccine (November 2022–March 2024).

Observation activities between November 2022 and March 2024	Estimated time spent (hr)	Related vaccines (where applicable)
Development of training materials and the delivery of training to managers and health providers	162	HPV, malaria, measles–rubella, rotavirus pneumococcal
Development of IEC materials	36	HPV, malaria, and measles–rubella
Development of monitoring and evaluation tools	12	All vaccines
NVIP planning meeting for introduction of typhoid conjugate vaccine	7	Typhoid conjugate
Stakeholders’ meetings (includes sensitizations or vaccine launch)	12	HPV, malaria
Malaria vaccine rapid assessment report writing workshop	16	Malaria
HPV vaccination performance review meetings with poor performing counties	23	HPV
One week measles support supervision at county, sub-county, facility, and community level	40	Measles–rubella
Meeting on how to integrate COVID-19 vaccination into routine immunization programme	3	COVID-19
NVIP Technical Working Group meetings	5	Cross-cutting^a^
National immunization programme progress review involving NVIP, partners, and county health management teams	5	Cross-cutting
NVIP review and update of the programme’s operational immunization training modules for health managers and health workers	40	Cross-cutting
Gavi funding portfolio planning and budgeting workshops	30	Cross-cutting
Gavi–NVIP partnership Workshop: progress review, funding feedback, and opportunities	8	Cross-cutting
Interagency coordinating committee meeting	6	Cross-cutting
KENITAG meeting	12	HPV, malaria
Total observation time across all observation activities	417	

^a^Cross-cutting refers to discussions that entailed various issues that were not necessarily tied to a specific vaccine.

Many of the observation events detailed in Table 1 were not directly tied to the focal study cases but rather provided contextual insights into the decision-making within NVIP and KENITAG. The observation guide captured key characteristics of the meetings, including the venue and purpose, the actors involved, and the nature of the discussion process and its outcomes (supplementary file S5, see online supplementary material).

Additionally, we used a document review guide (supplementary file S6, see online supplementary material) to extract information from a range of global and national documents to understand local evidence use practices, and how these practices were shaped by global guidance [Table 2 summarises the document categories and data extracted, while supplementary file S7 (see online supplementary material) lists the documents reviewed]. We identified relevant documents through targeted online searches in websites (e.g. WHO and MOH websites) and from NVIP offices, while additional materials were identified via references mentioned by study participants.

**Table 2:** Categories of documents and descriptions of data extracted.

Document category	Description of data extracted
Global guidance	Normative statements related to evidence use in vaccine policy decision-making (e.g. from Gavi funding guidelines or WHO position papers)
Legislative and policy documents	National guidelines related to evidence use in vaccine policy decision-making (e.g. KENITAG’s internal procedures manual)
NVIP and KENITAG reports and plans	Meeting minutes, vaccine introduction plans, funding applications, and programme assessments documenting actors, decisions, and evidence referenced in HPV and malaria vaccine policy decision-making
Training and communication materials	Health worker training packages and IEC materials related to HPV and malaria vaccines illustrating evidence use
Media	Media briefings and coverage highlighting public concerns, advocacy, and narratives related to vaccines and their policy environment.

Lastly, we conducted 34 in-depth interviews between January and October 2023. These interviews explored stakeholders’ roles in the introduction and roll-out of HPV and malaria vaccine as well as what, how, and why evidence is used in shaping these decisions (supplementary file S8, see online supplementary material). Interviews were conducted either face-to-face or online, depending on participant preference, and lasted between 40 and 120 min. Interviewees were purposively selected based on their previous experience and expertise in vaccine policymaking or their direct involvement in the policymaking process for HPV and/or malaria vaccines (Table 3). Initial participants were identified through the document review, followed by a snowballing approach to recruit additional respondents. Interviews continued until sufficient information had been gathered to address the research questions. As summarised in Table 3, interviewees included national MOH officials, KENITAG members and external consultants from local academic institutions, county EPI logisticians, representatives of international partner organizations, and local civil society organizations. We report participants by actor category and institutional role rather than individual nationality or race to protect anonymity, particularly because some categories included very few respondents, and because these characteristics were not systematically collected or analysed as demographic variables.

**Table 3:** Summary of the interviews conducted.

Range of actors	Role in vaccine policymaking	Numbers
National MOH officials	Vaccine policy formulation and coordinating roll-out activities	10
KENITAG members including external consultants from local academic institutions	Providing evidence-informed recommendations on vaccine policy introduction and roll-out	6
County EPI logisticians	Development of training materials for health workers, and overseeing the distribution of vaccines from national stores to facilities as well as immunization service delivery	3
International partner organizations	Financial and technical assistance for vaccine introduction and roll-out	13
Local civil society organizations	Community-based activities, such as advocacy, communication, and social mobilization	2
Total number of interviewees across all actor categories		34

### Data analysis

We utilized a framework approach to data analysis (Ritchie and Spencer 1994, Srivastava and Thomson 2009). This approach was applied across textual data from all data sources (i.e. observation notes, documents, and interviews). It involved five iterative analytical steps. First, we familiarized ourselves with the data by listening to audio-recordings and reading observation notes, document summaries, and interview transcripts. Second, we developed a coding scheme (supplementary file S9, see online supplementary material). The coding scheme provided an analytic structure for organizing and interpreting data, comprising categories and sub-categories that reflected the study’s focus on the types of evidence used, how evidence was used, and its influence relative to other factors, as informed by the study’s frameworks. Specifically, the evidence spectrum guided coding of evidence type and source, including whether evidence was scientific, tacit, or positioned between these categories, and whether it was local, regional/comparable, or global. Weiss’s models guided coding of how evidence was used and how it interacted with other factors, including instrumental, political, interactive, and imposed uses of evidence. Third, we went through all the data sources (observation notes, document review, and interview transcripts) thoroughly and manually linked relevant findings to corresponding themes from the coding scheme in a deductive coding process. Fourth, we sorted and charted the data according to the coding scheme. Finally, we compared and interpreted findings within and across data sources to identify patterns, associations, and explanations that adequately respond to the study’s objectives. As part of this interpretive process, we developed the evidence spectrum figures presented in the Results section by mapping coded evidence types and sources onto the scientific–tacit and local–global dimensions. Placement along the scientific–tacit dimension was based on qualitative judgements about how the evidence was generated, documented, and used, including the extent to which it reflected systematic processes or tacit professional, programmatic, or community knowledge. Placement along the local–global dimension was based on the degree of contextual specificity to the Kenyan vaccine decision-making setting. The resulting figures show the range of evidence inputs informing decisions or considerations across the three vaccine policymaking stages. Their exact positioning should therefore be interpreted as relative and analytical, rather than as a precise measurement. Preliminary findings were then collectively reviewed by the research team, including in-country health policy and systems experts (B.T., E.B., and J.N). This collaborative process helped refine analytical insights, validate assumptions, and strengthen the depth of descriptions and explanations.

## Results

Here, we present findings structured across three vaccine policymaking stages: vaccine prioritization (deciding whether to introduce a new vaccine for routine use in the country); vaccine policy formulation (deciding the detailed vaccine programme design features); and vaccine roll-out (operational/day-to-day decisions and activities supporting vaccine delivery). Although presented separately for analytical clarity, these stages overlapped in practice. For example, decisions about whether to introduce a vaccine often involved early consideration of programme design issues. Box 1 provides an overview of how the HPV and malaria vaccine policymaking processes unfolded. For each vaccine case, it moves from early discussions about introduction, to programme design, and then to roll-out activities.

Box 1 Overview of how the HPV and malaria vaccine policy processes unfolded

**Table:** 

**HPV vaccine** NVIP discussions about HPV vaccine introduction in Kenya began around 2011, following wider global policy and financing developments. WHO had published its first HPV vaccine position paper in 2009, and Gavi added HPV vaccine to its funding portfolio in 2011. Since countries such as Kenya lacked prior experience delivering multi-dose HPV vaccination to adolescent girls, Gavi required an initial pilot to generate evidence that could inform a later application for national introduction support. NVIP implemented a Gavi-supported HPV vaccine pilot in Kitui County from 2013 to 2015. These early HPV vaccine processes were already underway before KENITAG core members were appointed in 2014 and before KENITAG became fully operational in late 2015. Upon request from NVIP in 2017, KENITAG reviewed evidence, deliberated, and recommended national HPV vaccine introduction. Programme design was then taken forward through NVIP-led working group processes, informing the vaccine introduction plan, budget, and Gavi application submitted in 2017. Once Gavi funding had been secured, NVIP thematic sub-committees led roll-out activities, and HPV vaccine delivery began nationally in 2019. **Malaria vaccine** NVIP discussions about malaria vaccine introduction in Kenya were initially linked to the WHO-coordinated malaria vaccine pilot implementation programme. In 2015, WHO invited expression of interest to participate in the pilot from ministries of health in sub-Saharan Africa. Kenya expressed interest and was confirmed as a pilot country in 2017, with the malaria vaccine pilot launched in selected counties in September 2019. Following WHO’s recommendation for broader malaria vaccine use beyond pilot areas and Gavi’s addition of the malaria vaccine to its funding portfolio, NVIP requested KENITAG in 2022 to review available evidence and provide a recommendation on routine introduction. KENITAG subsequently recommended introduction beyond pilot areas. Programme design was then taken forward through NVIP-led working group processes during routine introduction planning, informing the vaccine introduction plan, budget, and Gavi application submitted in 2022. Once Gavi funding had been secured, NVIP thematic sub-committees led roll-out activities, with malaria vaccine delivery beyond the pilot areas starting in 2023.

For each vaccine policymaking stage, we applied the evidence spectrum approach to identify the different types of evidence used along the framework’s two dimensions: the scientific–tacit dimension and the local–global dimension. In the following subsections, we first describe evidence use within each stage and then interpret these patterns using Weiss’s evidence-use models. Table 4 provides a complementary summary of the key findings across the policymaking stages.

**Table 4:** Summary of key findings as highlighted by the application of the study’s frameworks.

Vaccine policymaking stage and key outcome	Evidence use practices and influencing factors	Evidence use model
Vaccine prioritization Decision on whether to introduce new vaccine	NVIP decision-making was hugely influenced by global guidance (i.e. WHO position papers) and access to global funds for vaccine introduction and roll-out. KENITAG prioritized evidence on the public health outcomes of vaccines over evidence on other socio-political considerations. Predominance of biomedical and public health experts in KENITAG influenced the above prioritization of evidence on public health outcomes.	Political and imposed
Vaccine policy formulation Detailed design of vaccine programme	NVIP’s HPV and malaria vaccine working groups predominantly used global guidance and local tacit evidence. Global directives in some policy design decisions elevated the use of scientific evidence over tacit considerations. Predominance of biomedical and public health experts in NVIP influenced the prioritization of evidence on public health outcomes of vaccines over evidence related to socio-political considerations	Imposed and interactive
Vaccine roll-out Operational decisions and activities discussed in Table 2	NVIP’s sub-committees predominantly relied on local evidence to inform operational decisions The use of scientific evidence in these decisions was initiated and supported by non-government actors (international partners or civil society organizations) Civil society evidence fostered acceptability of IEC materials and vaccines by integrating local socio-cultural values and trust-building narrative framing into advocacy and communication	Interactive, instrumental, and political

### Vaccine prioritization

NVIP’s decisions to introduce new vaccines, including HPV and malaria vaccines, were influenced by global guidance from WHO and by the availability of global funding sources. For example, some interviewees emphasised the importance of WHO position papers for both the HPV and malaria vaccines’ prioritization. Others pointed to Gavi funding as the primary driver of NVIP’s and international partners’ decisions to introduce new vaccines. This interpretation was echoed by some local experts within KENITAG, who perceived their involvement in evidence synthesis as largely intended to endorse NVIP’s funding interests and align with Gavi’s requirements, rather than to provide substantive local input on whether and how new vaccines should be introduced in the public sector.

‘Sometimes I have a feeling that the ministry already had a way they were going to get the money [to introduce the vaccine] from somewhere and [so they] just want to hear our opinion and unless we become completely negative, they were willing to go ahead.’ (DI29_2023, KENITAG)

Together, these accounts suggest that vaccine prioritization reflected ‘political’ and ‘imposed’ models of evidence use. It reflected a political model because evidence synthesis and KENITAG endorsement were perceived by some local experts as supporting an already preferred vaccine introduction and funding pathway. It reflected an imposed model because Gavi funding requirements, including the need for a KENITAG recommendation, shaped what evidence was needed and when it was mobilized and used.

When comparing the two vaccine introduction processes, interviewees noted that the HPV vaccine introduction began earlier than that of the malaria vaccine, with planning and discussions initiated before the establishment of KENITAG (Box 1). As a result, KENITAG had limited time and influence in shaping the HPV vaccine decision. Over time, however, KENITAG’s involvement and authority in vaccine decision-making expanded. By the time the malaria vaccine was under consideration, KENITAG had become more established, benefiting from greater donor support that allowed for more meetings over an extended period and the hiring of an expert in evidence synthesis to review available evidence and organize it using KENITAG’s evidence-to-decision framework. This institutional growth is reflected in the more comprehensive use of evidence for the malaria vaccine recommendation compared to the HPV vaccine (Fig. 2).

**Figure 2:**
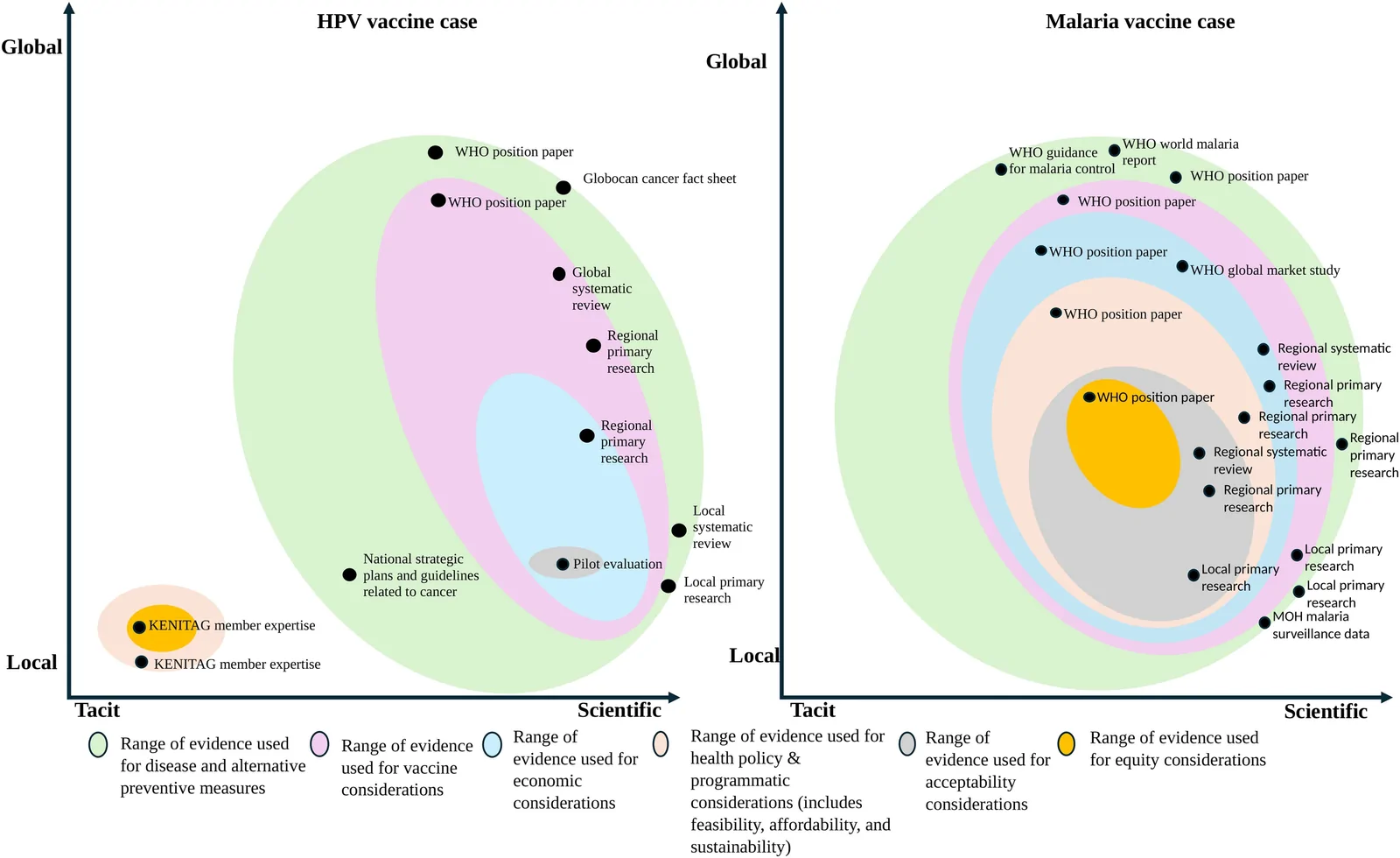
Range of evidence used by KENITAG’s working groups for HPV and malaria vaccine programme discussions.

As further illustrated in Fig. 2, KENITAG discussions about new vaccine introductions prioritized public health outcomes of vaccines by largely focusing on evidence of disease burden and vaccine effects (safety, efficacy, effectiveness), while evidence on socio-political considerations, including programmatic, feasibility, and acceptability considerations played a more limited role in informing the decision at this stage. One influential expert involved in KENITAG's malaria vaccine discussion attributed this evidence prioritization to their biomedical and public health professional backgrounds (supplementary file S3).

‘[For example] I am a paediatrician and a clinical epidemiologist. And I do my research work in [XXXXX]. That’s what I do so I can only speak towards that kind of evidence, of course recognizing there is the other sorts of evidence which are not necessarily my area of expertise….’ (IDI26_2023, KENITAG)

### Vaccine policy formulation

Once the decision to introduce the vaccine had been made, NVIP faced a series of additional decisions related to the vaccine programme’s design. These included decisions about vaccine delivery strategies (e.g. in schools or health facilities), geographical settings and target populations (which parts of the country and which age groups to prioritize), administration site and schedule (i.e. where on the body to administer the vaccine, at what age, and at what intervals), and vaccine brand preferences. These decisions were made by NVIP’s HPV and malaria vaccine working groups, comprised mainly of MOH officials and international partners with biomedical and public health expertise. Similar to KENITAG, this professional composition influenced a consistent prioritization of evidence on public health outcomes (e.g. disease burden) over socio-political considerations (e.g. ethical or community concerns).

‘Being a health programme, most of us [in the NVIP working groups] are trained, and have a lot of expertise in medical issues, not on how to… elicit needs of the community.’ (DI11_2023, MOH)

While multiple programme design decisions were made, this section focuses on three—geographical setting, vaccine brand preferences, and administration site and schedule—to illustrate the range of evidence types drawn upon and the models of evidence use that characterized the decision-making process in this stage. Figure 3 summarizes the working groups’ predominant use of global guidance and local programmatic expertise in their programme design decisions. Across these decisions, the use of scientific evidence was often tied to global technical and financial directives.

**Figure 3:**
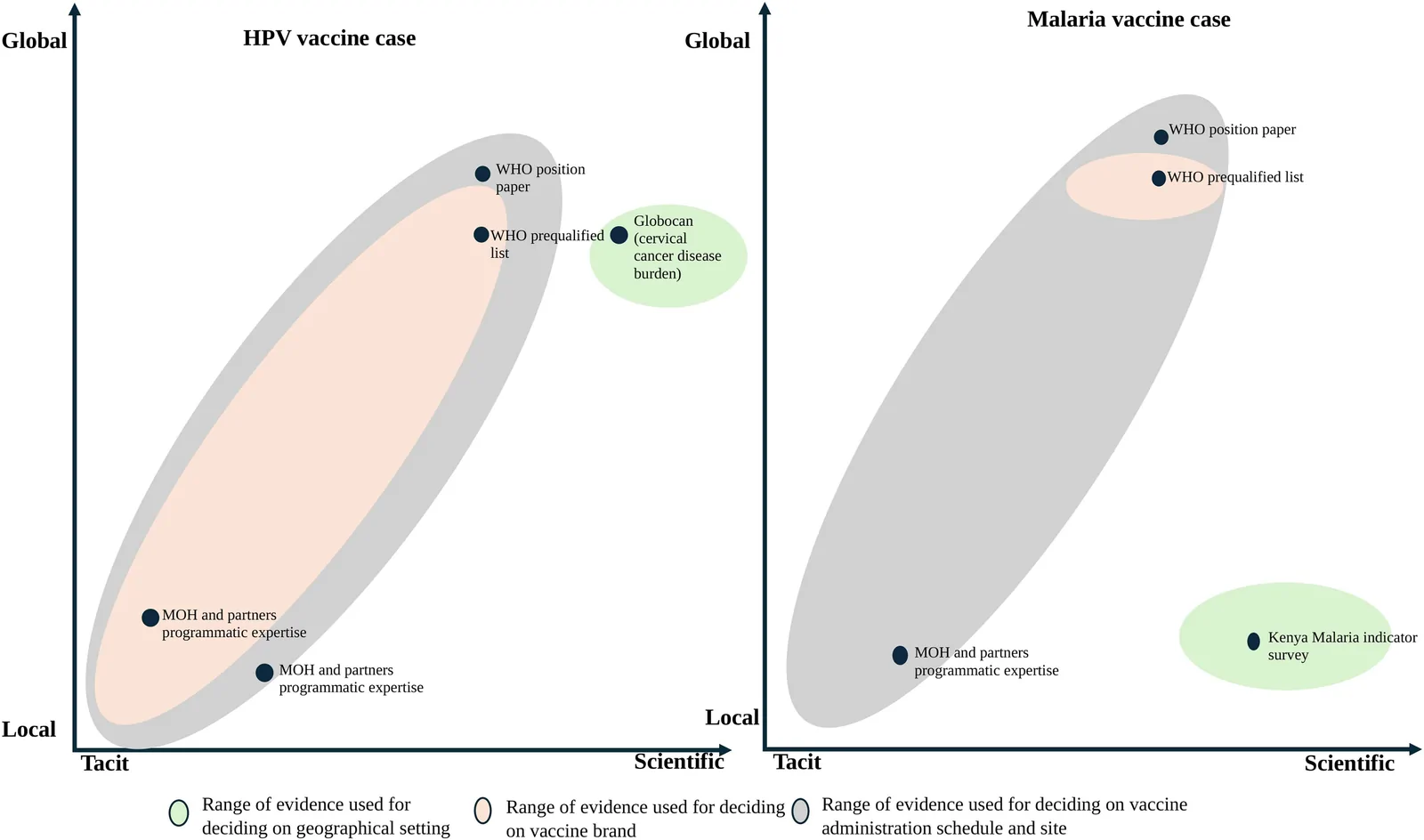
Range of evidence used by NVIP’s working groups for HPV and malaria vaccine programme design features.

Across both vaccine cases, the ‘imposed’ model reflected global technical and/or financial directives prescribing what types of evidence should be used, rather than nationally determined evidence generation and use. For instance, during Kenya’s malaria vaccine pilot in 2019, WHO recommended prioritizing sub-national regions experiencing moderate to high malaria transmission. Similar geographical targeting criteria applied during the later routine introduction in 2023, where they were reinforced by Gavi funding requirements and WHO’s 2022 framework for allocating limited malaria vaccine supply. This focus on transmission intensity appeared to have elevated the use of local scientific evidence on disease burden as the dominant basis for geographical site selection, limiting the weight of local ethical concerns about fair benefit-sharing with vaccine trial communities and political sensitivities around regional and ethnic representation (Box 2).

Box 2 Ethical and political tensions in deciding on malaria vaccine geographical targeting

**Table:** 

During the malaria vaccine pilot design, the selection of pilot counties based on epidemiological evidence generated important ethical and political debates that underscored how international actors’ requirements constrained local evidence use. Local ethical concerns emerged around the exclusion of Kilifi County, which had hosted the clinical trials for the malaria vaccine. Some stakeholders argued that Kilifi communities had a moral claim to benefit from continued access to the vaccine beyond the trials. However, the working group prioritized the potential public health benefits of targeting high disease burden regions thereby overlooking certain local views. As one international partner explained: ‘*Kilifi at that time was going to low to moderate [malaria transmission], of course with pockets of high disease burden. Those were some of the contentious heated discussions. So, probably an ethicist would have liked to roll it out in Kilifi and look good and feel good and make everyone happy, but we would have missed the key points that WHO wanted.*’ (DI07_2023, International partner) Local political tensions also surfaced within the selected malaria endemic lake region. A total of 12 out of 63 sub-counties in the malaria endemic lake region had been excluded due to lower malaria prevalence or ongoing malaria vaccine studies that could interfere with the pilot evaluation. Some local political leaders questioned this decision, however, viewing it as politically or ethnically motivated. For instance, in one county, all sub-counties selected for the pilot were affiliated with the opposition to the incumbent county governor, provoking political contestation over the perceived bias in selection (IDI07_2023). In another instance, the exclusion of two sub-counties with low malaria prevalence, and predominantly inhabited by one ethnic group, was locally perceived as a continuation of historical ethnic marginalization and therefore contested by the local politicians (IDI07_2023).

Therefore, NVIP’s malaria working group used findings from the Kenya Malaria Indicator Survey (Fig. 3) to select counties in the malaria-endemic lake region. This was the only region among the country’s epidemiological zones that met the funding criteria of moderate to high malaria transmission.

Similarly, for the HPV vaccine, the Gavi funding application required countries to provide evidence of the national cervical cancer disease burden from either country assessments or the Global Cancer Observatory (Globocan) (WHO 2024) as a justification for a national-level HPV vaccine introduction. Unlike malaria, which had been a longstanding priority health issue for the MOH, national surveys on cancer disease burden were not routinely conducted at the time of HPV vaccine introduction. Hence, findings on the national cervical cancer disease burden from Globocan (Fig. 3) were reportedly used by NVIP’s HPV working group to meet the directives of the donor, suggestive of the ‘imposed’ model.

Global technical and funding directives also shaped vaccine brand and procurement decisions, but in different ways across the two vaccine cases. LMICs using United Nations Children's Fund as the procurement entity, could not procure vaccines that were not on WHO’s prequalified list, a global guidance listing vaccines that have been evaluated and found to adhere to WHO defined standards for vaccine quality, safety, and efficacy (WHO 2023). For the malaria vaccine, this procurement requirement coincided with the fact that RTS,S was the only malaria vaccine that had completed clinical development and received WHO recommendation at the time, meaning no other alternative candidate was available for comparison. The ‘imposed’ model was therefore reflected in a constrained procurement context, where Gavi-supported procurement depended on WHO prequalification (Fig. 3), but the absence of competing malaria vaccine products meant there was no substantive brand-selection decision.

In contrast to the malaria vaccine, the HPV working group could choose between two WHO-prequalified vaccines (Fig. 3) within Gavi’s funding portfolio (the bivalent and quadrivalent HPV vaccines) (NVIP 2017a, 2017b). In this case, decision-making reflected both ‘imposed’ and ‘interactive’ models of evidence use. The imposed model was reflected in the restriction of vaccine brand options to WHO-prequalified products within Gavi’s funding portfolio. The interactive model was reflected in how multiple forms of evidence and judgements shaped the final choice between the bivalent and quadrivalent vaccines. For example, the HPV working group drew on their ‘biomedical and public health expertise’ (Fig. 3) to prioritize the quadrivalent vaccine, despite its higher demand on cold chain storage requirements, because it offered additional local public health benefits (i.e. protection not only against HPV types 16 and 18 associated with cervical cancer, but also against types 6 and 11, which cause genital warts) (NVIP 2017a, 2017b). This choice was further strengthened by working group members confidence in their ‘local programmatic expertise’ (Fig. 3) in managing storage challenges through securing donor support to address cold chain gaps and by optimizing vaccine delivery schedules (e.g. adjusting the timing or frequency of distributions based on storage capacity) (NVIP 2017a, 2017b).

The ‘interactive’ model of evidence use was also evident in how global guidance was not only adopted but also actively adapted through the integration of local, tacit evidence to better fit the country’s specific context. Here, tacit evidence consisted mainly of programme actors’ experiential knowledge of how immunization services are organized, how vaccination schedules are implemented, and how adverse events are monitored in routine practice. Starting with the HPV vaccine, the working group adopted WHO position paper’s (Fig. 3) recommendations to administer the vaccine in the deltoid muscle of the upper arm with a 6-month interval between doses (WHO 2014). Additionally, they used their ‘local programmatic expertise’ (Fig. 3) to contextualize this WHO recommendation by further specifying that the vaccine should be administered in the deltoid muscle of the right arm. This programmatic preference was reportedly due to the desire to avoid the left arm, as it was already designated for administering the tetanus vaccine in women of childbearing age (which includes school-going children eligible for the HPV vaccine). As such, they felt that administering both vaccines in the same arm might make it programmatically difficult to monitor vaccine adverse events for both HPV and tetanus vaccines during campaigns.

Similarly, the malaria working group combined WHO guidance with local programmatic expertise to adapt the vaccination schedule and administration site for the malaria vaccine. A WHO position paper (Fig. 3) had recommended intramuscular administration of the vaccine, with the first dose given around 5 months of age, the third dose completed by 9 months while maintaining a minimum interval of 4 weeks between the initial three doses, and a 15 to 18 months interval between the third and fourth doses (WHO 2018). Therefore, given their ‘local programmatic experiences’ (Fig. 3) that aligning vaccination schedules fosters vaccine uptake, they decided that the malaria vaccination schedule would be at 6, 7, 9, and 24 months of age. This was to align with vitamin A supplementation (provided at 6 and 24 months) and the first dose of measles–rubella and yellow fever vaccines (administered at 9 months) (NVIP 2017c, 2018, MoH 2023). Further, similar to the HPV vaccine, they used their ‘local programmatic expertise’ (Fig. 3) to select the left deltoid region for vaccine administration, as it was a site that was not being used for administration of other routine infant vaccines, to allow for effective programme monitoring of adverse effects related to the malaria vaccine (NVIP 2018).

### Vaccine roll-out

Following policy formulation, NVIP advanced to the vaccine roll-out stage, involving operational decisions and activities to support the start of vaccine delivery. These included developing Information Education and Communication (IEC) materials, developing training materials for health managers and health workers, microplanning, distributing vaccines and related supplies, and developing monitoring and evaluation tools. This section focuses on two key decisions, the development of IEC materials and microplanning and distribution, to illustrate the evidence types and models of use applied in this stage. Figure 4 highlights the predominant use of local tacit evidence in these decisions by NVIP thematic sub-committees. As in the policy formulation stage, international partners influenced the use of scientific evidence in these decisions, while local civil society organization’s (CSO) contributions were most visible in relation to the development of IEC materials.

**Figure 4:**
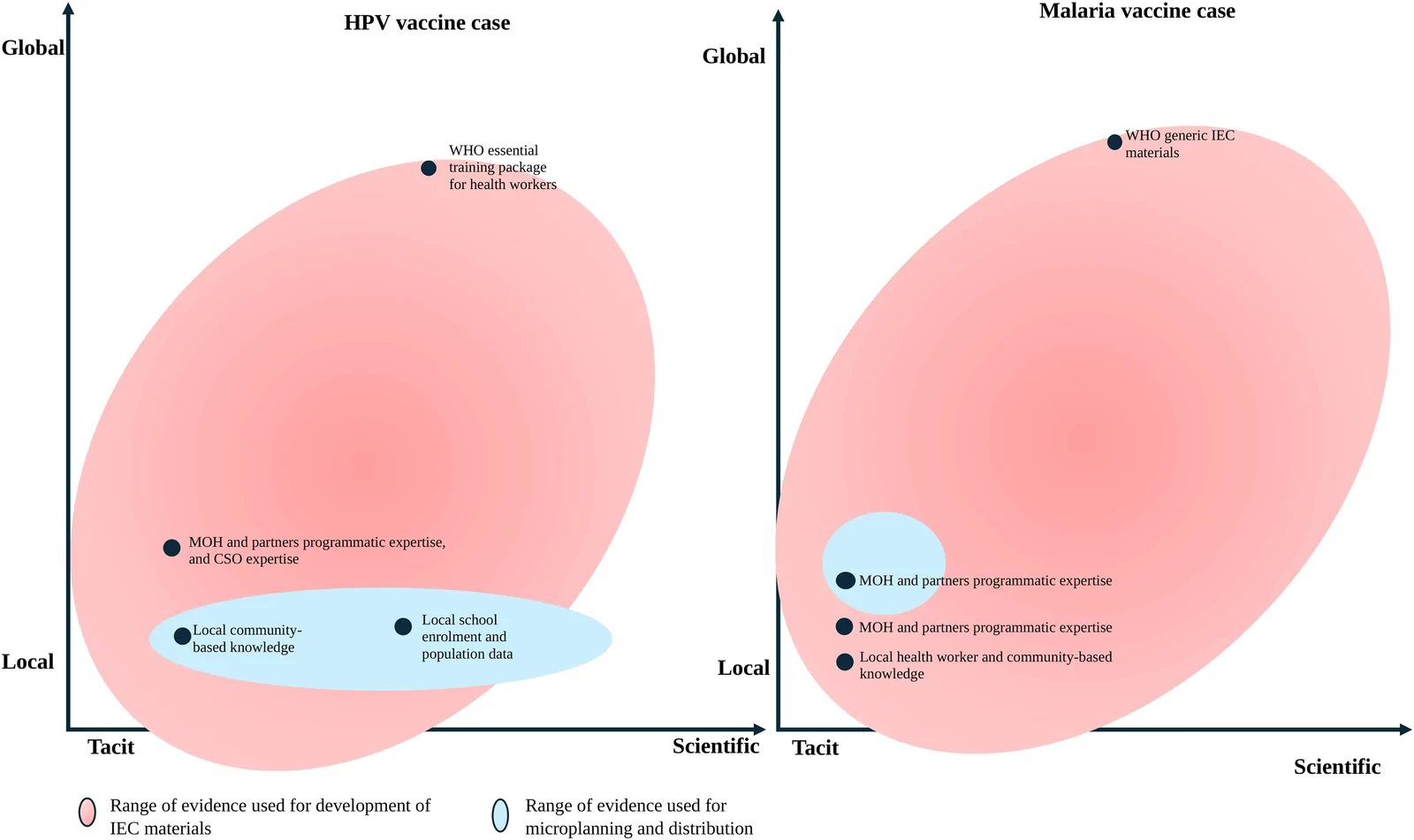
Range of evidence used by NVIP’s thematic sub-committees for HPV and malaria vaccine roll-out.

Across both vaccine cases, decisions on the development of IEC materials and strategies reflected ‘instrumental’ and ‘interactive’ models of evidence use. Starting with the malaria vaccine, international partners initiated and funded a local research study (Fig. 4) to explore health workers’ and the public’s views and values regarding the malaria vaccine in endemic regions. The study identified key IEC gaps (such as concerns about vaccine safety, effects, and affordability) which then informed WHO’s development of generic malaria vaccine IEC materials (Fig. 4) for health workers and the public. This phase reflected an instrumental model of evidence use, as findings from local research were directly used to develop generic IEC materials.

The subsequent local adaptation of WHO’s generic IEC materials introduced an interactive model of evidence use. The ACSM sub-committee actively adopted and adapted these materials drawing on local programmatic expertise, and health worker- and community-based knowledge (Fig. 4). At the national programmatic level, the generic materials underwent structural revisions to align with existing NVIP templates, while content was adapted to reflect national policies (such as free vaccination) and to ensure accessibility by using language that was understandable to non-clinicians.

At community level, international partners supported pre-testing of the IEC materials amongst health workers, community representatives, and caregivers in the targeted counties, which further enabled validation and enrichment of the materials through diverse sources of knowledge. The input of these community-based actors was useful in adapting IEC materials to reflect real-life contexts and socio-cultural values, fostering familiarity, trust, and positive sentiments that encouraged vaccine uptake. For instance, interviewees highlighted that community representatives wanted the IEC materials to better reflect the local population and their way of life by depicting people with darker skin tones, using local ethnic languages, and replacing the perfectly manicured green grass surrounding the homestead with a more natural setting—featuring a rough path and livestock grazing. On the other hand, health workers wanted the materials to include male figures taking children for vaccination to promote their involvement and ultimately better vaccine uptake.

For the HPV vaccine, an interactive model of evidence use was also apparent, as multiple types of evidence, including research, programmatic, and civil society expertise, were drawn upon in developing the IEC materials. A local CSO representative was invited to contribute to the ACSM sub-committee discussions. This civil society representative reported having drawn the content used for the development of the IEC materials from a ‘qualitative review on acceptability research studies’ (Fig. 4) that examined the knowledge, attitudes, and practices of local communities and health workers in relation to cervical cancer, screening, and HPV vaccination in Kenya. This work had been commissioned by her organization about a year before these discussions took place to support local awareness creation and advocacy on cervical cancer, screening, and HPV vaccination in Kenya. It identified, among other things, information gaps and needs from local communities and health workers as well as highlighting relevant and culturally appropriate communication strategies. The CSO representative described this contribution as bringing ‘knowledge of the community’ into the process:

‘*Some people will bring money, others will bring technical expertise, others will bring knowledge of the community. I brought knowledge of the community.*’ (IDI10_2023, CSO).

Afterwards, NVIP members reviewed and further modified the structure and content of the HPV vaccine IEC materials, informed by their local programmatic expertise (Fig. 4). For example, one of the tactics that they used to promote uptake of the vaccine was to frame it in the IEC materials as a cervical cancer prevention vaccine rather than HPV prevention vaccine to avoid talking about the fact that HPV is transmitted sexually. This was because they thought such a framing would be more acceptable to parents and religious leaders especially given that they were targeting young females. However, this framing was also emphasized in WHO’s HPV vaccine essential training package for health workers (Fig. 4), and this may have also influenced their decision-making.

Additionally, some of the interviewees praised the creative, persuasive, and/or emotive expert contribution of the civil society representative (Fig. 4) during the discussions on local advocacy strategies. The individual drew on local civil society knowledge of community advocacy and public trust-building to argue for an HPV vaccination narrative that could connect emotionally with the public and support acceptance. Specifically, they influenced the selection of the daughter of a cervical cancer survivor to be the first Kenyan girl to get the HPV vaccination in a widely publicised vaccine launch attended by the then president. The involvement of the president was also strategically intended to generate positive public emotion towards the vaccine, leveraging the president’s popular support to encourage widespread acceptance of the vaccine.

Unlike the malaria vaccine, there was no direct engagement or pre-testing of HPV IEC materials with healthcare workers and local communities to validate and enrich the materials. As a result, some of the content reflected a ‘national programme-centric’ perspective that assumed homogeneity across local contexts. This limited contextual adaptation reportedly undermined local ownership and the acceptability of both the materials and the vaccine in several marginalized settings. For example, when the HPV vaccine was rolled-out, most of the counties with low coverage were from where the socio-economic and cultural context is different from the rest of the country. Hence, county representatives argued that some of the reasons for their low vaccine coverage was that the IEC materials did not reflect their local values, e.g. by depicting girls without hijabs in predominantly Muslim areas and by omitting locally familiar imagery such as beekeeping, which is a key livelihood in some regions, instead using images of cattle grazing.

Unlike the above decisions regarding IEC material development, the logistics sub-committee’s decisions related to microplanning and distribution for the malaria and HPV vaccines demonstrated features aligned with instrumental and political models of evidence use, respectively. For the malaria vaccine, the choice to forego microplanning exemplified the instrumental model. The sub-committee used their local programmatic expertise (Fig. 4) to decide that microplanning was unnecessary because the malaria vaccine’s facility-based delivery strategy targeted children who were already visiting facilities for routine immunization services. Consequently, existing workflows could readily accommodate the malaria vaccine without requiring additional planning.

In contrast, microplanning was implemented for the HPV vaccine, engaging local actors such as facility representatives, community health volunteers, and teachers. These stakeholders utilized local community-based knowledge, population data, and school enrolment data (Fig. 4) to develop strategies aimed at reaching the target population. Despite this involvement, the process reflected characteristics of the political model of evidence use. Interviewees noted that the timing of microplanning was delayed, constraining the capacity of local actors to influence decision-making meaningfully. Critical resources for service delivery and community engagement had already been allocated during earlier stages of policy formulation, thereby limiting flexibility for local adaptation of strategies. As a result, some interviewees perceived microplanning as largely tokenistic, serving primarily as a procedural formality linked to financial allowances rather than as a substantive mechanism for local adaptation.

‘*If I am being honest, probably I should not say this, but the micro planning process is just an activity to fill in the books. Because it is an expensive activity, and it needs to be done on a very large scale. But nobody really looks at them. It is just an activity to be checked off and it is a money-making activity.*’ (IDI20_2023, International partner)

## Discussion

In this study of evidence use in vaccine policymaking in Kenya, we identified five major themes that we discuss in this section. First, international partners have shaped Kenya’s vaccine policymaking, by taking on financing and technical support roles across multiple policy stages. Thus, they were able to consistently ‘construct’ or ‘legitimize’ what constitutes priority vaccine issues and the evidence that should be used by national policymakers to inform vaccine policy. These roles and the influence they carry ultimately promoted the use of global guidance and scientific evidence, reduced evidence use to a procedural formality (or tick-box activity) that secures funding, and marginalized other local evidence use considerations and deliberations. Similar findings about the influence of international partners on national policy agendas and related evidence generation and use have been highlighted in other health policy studies done in Kenya (Oronje 2016) and other LMICs (Shiffman 2014, Khan et al. 2018, Storeng et al. 2019).

Second, professional expertise and the roles of those physically represented in policymaking spaces acted as cognitive filters shaping the type of evidence that they valued in vaccine decision-making. Hence, the dominant physical representation of biomedical and public health experts working in public health organizations contributed to the consistent prioritization of public health outcomes of vaccines over local socio-political considerations. This suggests that evidence authority was shaped not only by the formal availability of evidence, but also by who was present in decision-making spaces and what forms of knowledge they were professionally equipped to interpret and value. This aligns with wider literature highlighting that biomedical and public health professions often dominate health and vaccine policymaking, leading to decisions that may sideline alternative perspectives that are critical for ensuring contextually responsive, inclusive, and accountable policy decisions (Oliver and Pearce 2017, Ward et al. 2019).

Third, the study findings highlight the epistemic value of local tacit evidence i.e. local actors’ experiences, perspectives, and values in vaccine policymaking. Tacit evidence provided local operational and contextual insights that were instrumental in validating and contextualizing global guidance and scientific evidence. However, the use of local tacit evidence in earlier vaccine policymaking stages could have been strengthened through more deliberate engagement with experiential knowledge from affected and implementation actors, including caregivers, adolescent girls, teachers, religious and community leaders, health managers, health workers, and community health volunteers. Earlier incorporation of these forms of knowledge could have added depth and contextual specificity to existing considerations such as acceptability, feasibility, equity, and implementation. It could also have ensured that these considerations were informed not only by technical deliberation, but also by the lived and practical experiences of those affected by or responsible for implementing vaccine programmes. This aligns with wider literature recognising the legitimacy and importance of experiential and context-specific forms of knowledge in health-related decision-making, and cautioning against privileging scientific evidence in ways that marginalize other relevant forms of knowledge (Gabbay and le May 2004, Kothari et al. 2012).

Fourth, the study findings highlight the limited role and influence of civil society actors in vaccine policy decisions at the national level in Kenya, with their evidence contributions primarily informing IEC materials rather than broader vaccine policy or operational decision-making. The poor inclusion of civil society voices or evidence in other studies of vaccine policymaking in both LMICs (Burchett et al. 2012b) and high-income countries (Ward et al. 2019) suggests that their value remains underutilized in vaccine policy processes. In contrast, studies done in other health policy contexts have highlighted civil society participation and contributions as valuable in challenging policymakers’ assumptions and capturing local values, preferences, and needs that conventionally used evidence (e.g. clinical and quantitative data) do not fully address (Bernabe et al. 2014, Lopes et al. 2016, Leopold et al. 2020, Bidonde et al. 2021, Jacobs and George 2022). Some of these policy contexts include equally technical decision spaces such as health technology (or drug) assessment committees whereby consumer representatives shared rich, experiential insights that were crucial to understanding the impact of health conditions and appropriately weighting different types of evidence (Lopes et al. 2016).

Lastly, we found that applying both the evidence spectrum approach to categorizing evidence and Weiss models of evidence use was complementary. They enabled us to reflect continuously on the various types of evidence used and the diverse pathways through which evidence influences policy, beyond simplistic and linear relationships. Also, the spectrum approach views different types of evidence as equally valid sources, rather than being arranged in traditional hierarchies or pyramids that rank some forms of evidence above others. By allowing users to simultaneously consider both the nature of evidence and the geographical jurisdiction in which it is generated, this approach moves thinking beyond viewing scientific or tacit sources as fixed, bounded categories. Moreover, the visual representation along two axes may help to promote more nuanced and reflexive conceptualizations of what counts as evidence for decision-making, supporting ongoing calls to broaden the range of evidence deemed legitimate in health policymaking, and to foster more inclusive and accountable decision-making processes (WHO 2021).

In relation to application of the evidence use models, we made four key observations. First, the interactive, political, and imposed models were particularly useful for interpreting vaccine policymaking in a donor-influenced context where global guidance, Gavi funding requirements, and national technical deliberation were closely intertwined. The interactive model helped capture how multiple evidence types and actor judgements were considered together through deliberation among actors involved in vaccine policy processes, while the political and imposed models helped explain how evidence was mobilized to support preferred policy pathways and meet procedural or funding requirements. Second, although the Weiss (1979) models of evidence use can be viewed as rooted in a narrow conceptualization of evidence as research, these models were applicable to a broader conceptualization of evidence that includes tacit evidence. This enhances Weiss’s evidence use models’ applicability to health policy and systems decisions. Third, the overlapping application of evidence use models within the same decision or decision spaces highlights the value of viewing these models as heuristic tools for understanding the multiple ways evidence informs policymaking, rather than as rigid frameworks for categorizing evidence use. Fourth, it was often challenging to isolate and specifically examine the role of specific pieces of evidence (e.g. a particular research study) in specific vaccine decisions. Future research should explore the optimal level of decision-making space to which the evidence use models should be applied.

### Study limitations

The first limitation of this study is that we focused on two vaccine policy cases only. While it is likely that the findings extend to other areas of vaccine policymaking, further research is needed to explore the extent to which the findings extend to other health policies. Second, the study focused on evidence use in the formative stages of vaccine policymaking, from prioritization through policy formulation and initial roll-out, rather than the period after vaccine delivery had begun. Evidence use after roll-out may serve different purposes, including performance monitoring, evaluation, and policy learning or adaptation. This phase may also provide additional opportunities for community and health worker experience to inform national vaccine policy. Future research could examine the post-roll-out phase, including how evidence generated through routine monitoring, community feedback, and the experiences of health managers, health workers, community health volunteers, and caregivers is incorporated into the adaptation of vaccine policy and delivery. Third, beyond documentary analysis, the study did not directly capture the perspectives of politicians, health workers, or the public—groups that were outside our scope but whose perspectives warrant future study. Finally, the sensitive nature of policymaking may have led some participants to provide socially desirable responses or assumptions about the process. However, triangulating data from multiple sources and actor groups helped identify and reconcile potential biases, ensuring a more robust and credible analysis.

## Conclusions

This study emphasizes the need for a comprehensive and inclusive approach to evidence conceptualization, generation, and use in vaccine policymaking—one that ensures scientific rigour while also valuing diverse forms of local evidence including from civil society. Theoretically, it advances Weiss’s models of evidence use by demonstrating their applicability to a broader spectrum of evidence beyond formal research and by showing how these models, when combined with a novel evidence spectrum approach, enable more nuanced and reflexive understandings of how different evidence types influence policy. Practically, achieving inclusivity in evidence use requires the addressing of underlying power imbalances that shape what is recognized as credible evidence, especially those rooted in the funding and technical assistance relationships between donors and local actors. Reconfiguring these relationships to value and integrate diverse types of local evidence at all stages of the vaccine policymaking process can strengthen both the legitimacy, acceptability, and effectiveness of vaccine policy decisions.

## Supplementary Material

czag100_Supplementary_Data

## Data Availability

The data underlying this article include interview transcripts, observation notes, document extraction files, and reviewed policy and planning documents. The anonymised excerpts presented in the article and supplementary materials may be used with appropriate citation. The full qualitative dataset is not publicly available because it contains sensitive information from a small group of potentially identifiable vaccine policy actors and includes observations of policy and planning meetings, making complete de-identification difficult without compromising participant confidentiality and institutional permissions. Publicly available documents analysed in the study are cited in the article or listed in supplementary file S7 (see online supplementary material). Internal or non-public documents and de-identified qualitative data may be made available upon reasonable request through the KEMRI-Wellcome Trust Research Programme Data Governance Committee at dgc@kemri-wellcome.org. Access will be subject to relevant ethics and institutional approvals, the consent conditions under which the data were collected, and whether adequate de-identification can be achieved to protect participants, organisations, and other individuals mentioned in the data.

## Reflexivity statement

This study was undertaken by a multidisciplinary team of researchers collaborating under the Supporting Inclusive and Accountable Health Systems Decisions for Universal Health Coverage Project (SUPPORT-SYSTEMS). D.W., J.N., E.B., and B.T. are based at the KEMRI–Wellcome Trust Research Programme in Kenya, while S.L., U.G., and C.G. are affiliated with academic and public health institutions in Norway. The team brings expertise in health systems and policy research, health economics, and clinical and qualitative research, with several members working at the interface of evidence and policy in both high-income and low- and middle-income contexts. Throughout the research process, the team remained reflexive about how our positionalities shaped the research. Collaborating across Kenyan and Norwegian institutions, where some team members contribute to the development of global guidance documents, prompted continual reflection on the asymmetries between global and local forms of evidence authority. Regular cross-site discussions enabled us to interrogate our own assumptions, including about what constitutes credible evidence, and how these assumptions may have influenced interpretation. These reflexive exchanges helped ensure that we stayed attentive to participants’ perspectives and to the diverse ways in which evidence and context interact in shaping health policy decisions.
