# Supplementary material for: Examining evidence use in vaccine policymaking in Kenya: a qualitative case study
Source: Health Policy Plan. 2026 Aug 10;41(9):1511–26. doi: 10.1093/heapol/czag100 (PMC13678818; doi:10.1093/heapol/czag100)
Supplement: Supplementary file 1 [file czag100_supplementary_data.zip › Supp file 1.docx]

**Supplementary file 1: Timeline of introduction of new and routine vaccines in Kenya's national immunisation programme between 1970 and 2023, including, related WHO and Gavi global events that influenced the introductions**

| **1974 (WHO global event):** WHO established EPI focusing on vaccines against 6 diseases:  Pertussis, tuberculosis, diphtheria, tetanus, polio and measles. Additionally, yellow fever is recommended for endemic countries |  | **1992-1997 (WHO global event):** WHO recommended introductions of Hepatitis B vaccine and Haemophilus influenza type B vaccine |  | **2001 (Local event):** NVIP introduced Haemophilus vaccine, Hepatitis B vaccine, and yellow fever vaccine |  | **2008-2016 (Gavi global event):** Gavi started funding introductions of: pneumococcal conjugate vaccine (2008); rotavirus vaccine (2009); HPV vaccine (2011); inactivated polio vaccine (2013); Rubella vaccine as part of the combined measles-rubella (2014); and malaria vaccine pilots (2016) |  | **2019 (Local event):** NVIP introduced HPV vaccine and malaria vaccine pilot |  | **2022 (Gavi global event):** Gavi started funding introduction of malaria vaccine |  |
| --- | --- | --- | --- | --- | --- | --- | --- | --- | --- | --- | --- |
|  | **1980 (Local event)**: NVIP was established focusing on WHO’s EPI vaccines |  | **2000-2001 (Gaviglobal event):** GAVI was established, and started funding introductions of Hepatis B vaccine, Haemophilus vaccine, and yellow fever vaccine |  | **2007-2015 (WHO global event):** WHO recommended introductions of: pneumococcal conjugate vaccine (2007); HPV vaccine (2008); rota virus vaccine (2009); rubella vaccine (2011); inactivated polio vaccine as part of polio eradication and endgame (2013); and malaria vaccine pilot (2015) |  | **2011- 2016 (Local event):** NVIP introduced: pneumococcal conjugate vaccine-10 (2011); measles second dose (2013); HPV vaccine pilot (2013-2015); rota virus vaccine (2014); inactivated polio vaccine as part of polio eradication and endgame (2015); and switch from measles to measles-rubella vaccine (2016) |  | **2021 (WHO global event):** WHO recommended introduction of malaria vaccine |  | **2023 (Local event):** NVIP introduced malaria vaccine |
