# Supplementary material for: Examining evidence use in vaccine policymaking in Kenya: a qualitative case study
Source: Health Policy Plan. 2026 Aug 10;41(9):1511–26. doi: 10.1093/heapol/czag100 (PMC13678818; doi:10.1093/heapol/czag100)
Supplement: Supplementary file 1 [file czag100_supplementary_data.zip › Supp file 2.docx]

**Supplementary file 2: Definitions of Carol Weiss's models for research use** (Weiss, 1979, Weiss et al., 2005)

| Model | Description and key assumption |
| --- | --- |
| Knowledge driven | Research/evidence is used directly to develop new tools, drugs, technologies, or interventions. |
| Instrumental or problem solving | Research/evidence is directly applied to inform a decision or action. |
| Interactive | Research/evidence is used alongside other inputs, including stakeholder perspectives, institutional interests, and deliberation. In this study, this model was interpreted as cases where multiple evidence types and actor judgements were considered together through deliberation. |
| Political or symbolic | Research/ evidence is used to justify, support, or legitimise a fixed and predefined policy position rather than primarily to shape it. |
| Tactical | Research/evidence is used for strategic purposes (e.g., to demonstrate government responsiveness, deflect criticism, or delay action) rather than its substantive findings. |
| Enlightenment or conceptual | Research/evidence shapes the general concepts and perspectives that shape policymakers' understanding of social issues. |
| Intellectual enterprise of society | Research/evidence as an interconnected component of societal intellectual activities, akin to fields like history, journalism, or philosophy. |
| Imposed or enforcement | Research/evidence use is tied to external funding, regulatory, procedural, or compliance requirements.(Weiss et al., 2005). |
