# Supplementary material for: Examining evidence use in vaccine policymaking in Kenya: a qualitative case study
Source: Health Policy Plan. 2026 Aug 10;41(9):1511–26. doi: 10.1093/heapol/czag100 (PMC13678818; doi:10.1093/heapol/czag100)
Supplement: Supplementary file 1 [file czag100_supplementary_data.zip › Supp file 3.docx]

**Supplementary file 3: Kenya National Immunization Technical Advisory Group’s core members expertise and organizations they work**

Core members expertise

|  | Proportion |
| --- | --- |
| Paediatrics | 38.4615% |
| Infectious diseases | 7.69231% |
| Adult medicine | 7.69231% |
| Microbiology | 7.69231% |
| Vaccinology and pharmacy | 7.69231% |
| Immunology | 7.69231% |
| Epidemiology | 7.69231% |
| Health services management | 7.69231% |
| Medical and public health law | 7.69231% |

Core members organisations

|  | Proportion |
| --- | --- |
| The University of Nairobi | 41.6667% |
| Kenya Paediatric Association | 16.6667% |
| Kenya Medical Research Institute | 16.6667% |
| Kenya Pharmacy and Posion's Board | 8.33333% |
| Private practice | 8.33333% |
