# Supplementary material for: Examining evidence use in vaccine policymaking in Kenya: a qualitative case study
Source: Health Policy Plan. 2026 Aug 10;41(9):1511–26. doi: 10.1093/heapol/czag100 (PMC13678818; doi:10.1093/heapol/czag100)
Supplement: Supplementary file 1 [file czag100_supplementary_data.zip › Supp file 4.docx]

**Supplementary file 4: Kenya National Immunization Technical Advisory Group’s evidence-to-decision framework drawn from its internal procedures manual.**

| **Issue** | **Elements** | **Specific data to collect** |
| --- | --- | --- |
| 1. **Disease** | Burden of disease | - Incidence of infection and sub-populations (age, sex, and co-morbidity) with more severe forms of disease. - Disease occurrence over time. - Epidemic potential. - % of infected becoming carriers and risks factors. - Short- and long-term consequences of infection and frequency. - Social impact of the disease. |
|  | Use and costs of health care | - Short- and long-term use of health care (incl. treatments and hospitalization) - School and work absenteeism |
|  | Alternative preventive measures | - Alternative preventive measures (e.g, health education, better hygiene, vector control) and their effectiveness, costs, and practicality - Other existing vaccines against the same disease and their effectiveness, costs, and practicality |
| 2. **Vaccine immunisation characteristics** | Vaccine presentation and use | - Vaccine presentation, storage volume and cold chain requirements - Dosage and route of administration - Administration schedule and possibility of combination with other vaccines - Flexibility or alternative vaccination schedules to accommodate the present national immunuzation programme’s schedule |
|  | Vaccine indirect effects | - Herd immunity - Impact on strain selection |
| 3. **Vaccine intervention outcome specific data** | Safety | - Type, consequences and frequency of short- and long-term adverse events following vaccination. - Type and frequency of risk groups or risk factors for adverse events. - Contra-indications for vaccination. - For live attenuated vaccines: risk of reversion to virulence. |
|  | Efficacy and Effectiveness by population and /or subgroups of population | - Type-specific protection afforded. - Critical determinants of the immune response associated with protection. - Duration of protection - Waning immunity if any - Optimal vaccination schedule (dosage, age, and booster) to protect the vaccinated individual |
| 4. **Economic considerations** | Disease related costs | - Direct and indirect costs to patients and families - Productivity losses |
|  | Vaccine related costs and resource use | - One-time start-up costs to implement the vaccine (i.e., cold chain investments) - Annual incremental recurrent costs to administer the vaccine - Costs to monitor safety and effectiveness of the vaccine |
|  | Net impact of intervention on immunization program as well as health sector | - Reduction in health care costs - Years Lived with Disability ; Disability- Adjusted Life Years or Quality-adjusted life years - Cost-effectiveness ratio of vaccination program |
| 5. **Health policy and programmatic issues** | Interaction with other existing intervention and control strategies | - Impacts of program (catch-up) on safety and efficacy of other vaccines and other health care sectors |
|  | Feasibility | - Availability of the vaccine and long-term supply - Accessibility of target population |
|  | Affordability and sustainability | - Availability of long-term human, technical and financial resources for distribution (including cold chain stability) - Partnerships |
|  | Ability to evaluate | - Availability of information systems to measure coverage and vaccine utilization. - Reliability of surveillance system |
|  | Regional and international considerations | - Existing of regional and global recommendations - Potential of disease for international spread and pandemic potential |
| 6. **Acceptability and equity** | Acceptability | - Perception of the public and medical community about the disease |
|  | Equity | - Universality, accessibility and gratuity of services for the most vulnerable groups |
