# Supplementary material for: Examining evidence use in vaccine policymaking in Kenya: a qualitative case study
Source: Health Policy Plan. 2026 Aug 10;41(9):1511–26. doi: 10.1093/heapol/czag100 (PMC13678818; doi:10.1093/heapol/czag100)
Supplement: Supplementary file 1 [file czag100_supplementary_data.zip › Supp file 5.docx]

**Supplementary file 5: Observation guide**

**Date of observation:**

**Venue of observation:**

**Meeting purpose:**

**Start time:**

**End time:**

1. Observe which actors are in attendance and their diversity (e.g., roles and professions)
2. Observe which actors are absent and their diversity?
3. Observe what roles various actors play? (Listen to actor introductions)
4. Observe who chairs the meetings?
5. How does the meeting chair foster attendees to openly discuss issues?
6. Are there actors agreeing or disagreeing on an issue relevant to study cases?
7. How are the actors relating to each other during and after meetings?
8. Who has the final say during meetings?

9. What venue is used for meetings and how are the seating arrangements?
10. Are the goals of the meeting made clear at the beginning of the meeting?
11. Is the meeting open or closed to the public and other stakeholders?
12. Is there detailed recording or real-time reporting of decision-making procedure or does the recordings or reporting take place after the decision-making has concluded?

13. Observe what informs discussions and decisions made
14. Observe the process of making final decisions (voting or consensus or both)
15. Observe how the meetings end including any communications regarding access to the meeting minutes and reports
