# Supplementary material for: Examining evidence use in vaccine policymaking in Kenya: a qualitative case study
Source: Health Policy Plan. 2026 Aug 10;41(9):1511–26. doi: 10.1093/heapol/czag100 (PMC13678818; doi:10.1093/heapol/czag100)
Supplement: Supplementary file 1 [file czag100_supplementary_data.zip › Supp file 6.docx]

**Supplementary file 6: Document review guide**

**Title:**

**Author:**

**Publication date:**

**Type of document:**

**Purpose of document:**

1. Is there reference to the term evidence and related terminologies (such as science, research, data, knowledge and experiences) in the document?

2. Is there description of how evidence (and related terminologies) ought to be used and governed in public health sector decision-making and vaccine policymaking?

3. How is the formal vaccine policymaking process in Kenya structured, particularly in terms of evidence conceptualization, utilization, and governance?

- How does the actual decision-making process, including evidence conceptualization, utilization, and governance, in the selected vaccine policy cases compare to the formal vaccine policymaking framework in Kenya? (check meeting minutes)
- Are there any concerns or contestations regarding the specific vaccine policy cases

4. Who are the key actors involved in vaccine policymaking in Kenya and what are their respective roles and reporting channels?

- Who were the specific actors engaged in the selected vaccine policy cases, and what roles did they play in the decision-making process?
- Were there any actors who should have been involved in the vaccine policy cases but were excluded? If so, what factors contributed to their exclusion?
- Are there any statements/references to when/how frontline health providers preferences are considered in the formal and actual vaccine policymaking process?
- Are there any statements/references to when/how public preferences are considered in the formal and actual vaccine policymaking process?

5. What contextual factors (historical, socio-cultural, and political) shape vaccine policymaking in Kenya, particularly in relation to evidence use? For example:

- Which key actors and factors have shaped the evolution of vaccine policymaking in Kenya over time, particularly in relation to evidence use?
- Is there a dedicated budget line for vaccine policymaking, including processes related to evidence generation and use?
