# Supplementary material for: Examining evidence use in vaccine policymaking in Kenya: a qualitative case study
Source: Health Policy Plan. 2026 Aug 10;41(9):1511–26. doi: 10.1093/heapol/czag100 (PMC13678818; doi:10.1093/heapol/czag100)
Supplement: Supplementary file 1 [file czag100_supplementary_data.zip › Supp file 7.docx]

**Supplementary file 7: List of the various types of documents reviewed**

| **Type of documents** | Non-exhaustive list |
| --- | --- |
| Legislative documents | - Kenya's Constitution of 2010 - The Science and Technology Act of 2009 - Science, Technology and Innovation Act of 2013 - Health Act of 2017 |
| Policy documents | - Kenya Health Policy of 1994 - Kenya Health Policy of 2014 to 2030 - National Health Sector Strategic Plan of 2005 to 2010 - Kenya Health Sector Strategic Plan of 2013 to 2017 - Kenya Health Sector Strategic Plan of 2018 to 2023 - Ministry of Health (MOH) Guidelines for Evidence Use in Health Policymaking in Kenya, 2016 - MOH Guidelines for Monitoring and Evaluation - National Policy Guidelines on Immunisation, 2013 - National Policy Guidelines on Immunization, 2023 - Kenya National Immunization Technical Advisory Group Internal Procedures Manual - Terms of references for the National Vaccine’s and Immunization Programme’s technical working groups and inter-agency coordinating committee - Kenya Expanded Programme on Immunisation draft plan of operation 1991-1995 - Concept paper for the establishment of a National Immunisation Technical Advisory Group for Kenya - Re-launching the National Immunization Technical Advisory Group (NITAG) establishment process in Kenya: Short report on Supporting Independent Immunisation and Vaccine Advisory Committees in East and Southern Africa (SIVAC) initial meeting with Kenya MOH. - Re-launching the NITAG establishment process in Kenya: Short report on SIVAC's initial meeting with Kenya MOH. |
| WHO documents (cited in policy documents or by interviewees) | - Global Immunization Vision and Strategy 2006-2015 - Global Vaccine Action Plan 2013 - World Health Assembly 2012 - Vaccine introduction guidelines. Adding a vaccine to a national immunisation program: decision and implementation 2005 - National Immunization Technical Advisory Groups: Guidance for their establishment and strengthening. |
|  | - WHO position papers for Human Papilloma Virus (HPV) vaccines - WHO position papers for malaria vaccine - WHO list of prequalified vaccines - WHO considerations regarding consent in vaccinating children and adolescents between 6 and 17 years old - WHO essential training packages for health workers - WHO HPV vaccine coverage monitoring tool |
| NVIP and KENITAG’s meeting reports | - Meeting minutes of the National Vaccines and Immunization Programme Technical Working Groups (includes for HPV and malaria) and thematic sub-committees discussions related to HPV and malaria vaccine - Meeting minutes of Kenya National Immunization Technical Advisory Group discussions related to HPV and malaria vaccines - Meeting minutes of interagency coordinating committee discussions related to HPV and malaria vaccines - Meeting minutes of Gavi, the Vaccine Alliance official visits - Stakeholder consultative meeting on HPV introduction and safety |
| NVIP’s programme assessments and reports | - Integrated post-introduction evaluation for HPV and malaria vaccines - Baselines study on knowledge, attitude, beliefs, and practices on HPV vaccine uptake in Kenya - Cost-Effectiveness Analysis of Routine Delivery of RTS,S/AS01 in Kenya, Malawi, and Ghana After 18 to 24 months of pilot implementation: Report to WHO and Gavi - Health Utilization Study in malaria vaccine implementation programme - Advocacy, communication, and social mobilization experiences and lessons during malaria vaccine implementation in Kenya |
| NVIP’s Gavi funding application forms | - Application Form for Country Proposals Providing two years of support for an HPV Vaccination Demonstration Programme - Application form for Gavi new vaccine support for HPV and malaria vaccines |
| NVIP’s new vaccine introduction plans | - HPV and malaria vaccine introduction plans - HPV micro plans - NVIP’s annual work plan |
| NVIP’s health managers and worker training materials | - NVIP’s training package for HPV vaccine introduction - NVIP’s training Package for Malaria vaccine introduction |
| NVIP’s Information, education, and communication materials | - Posters - Fliers - Banners - Flip charts - Leaflets (Medical professionals, religious leaders, teachers, women leaders) - Frequently Asked Questions - Key facts - HPV immunization card |
| Media articles | - Media briefings by Kenya Conference of Catholic Bishops and ongoing court cases against vaccination in schools |
