# Supplementary material for: Examining evidence use in vaccine policymaking in Kenya: a qualitative case study
Source: Health Policy Plan. 2026 Aug 10;41(9):1511–26. doi: 10.1093/heapol/czag100 (PMC13678818; doi:10.1093/heapol/czag100)
Supplement: Supplementary file 1 [file czag100_supplementary_data.zip › Supp file 8.docx]

**Supplementary file 8: Interview guide**

**Date of interview:**

**Venue:**

**Duration:**

**Interviewer code**:

1. Briefly introduce yourself, that is, your profession background, where you work and what it involves?

2. Before we start the main discussion, I have two questions to sort of set the scene for the discussion that follows:

- What is your understanding of what evidence is?
- What is your understanding of who relevant stakeholders are when making vaccine policy decisions?

3. How did you first hear about the introduction of HPV/ malaria vaccine in Kenya?

4. After you first heard about the HPV/malaria vaccine, what activities (meetings/ events) related to its introduction in the country followed? Probe on whether and how the participant was involved in any of the following activities and what informed their decision-making

- Design and implementation of a pilot
- Design of the new vaccine programme (i.e., vaccine delivery strategy, setting, target population, vaccine preference, administration, consent, and forecasting)
- Planning for vaccine roll-out (development of: training materials, monitoring and evaluation tools, microplanning and distribution, information, education, and communication messages and strategies)

5. What is the current status of HPV/malaria vaccine rollout and service delivery in the country? Any positives and challenges?

6. Reflecting on the introduction and rollout of the HPV/malaria vaccine policy:

- Which stakeholders had the most influence, and what factors contributed to their impact?
- Which stakeholders had unexpectedly low involvement or influence, and what factors may have contributed to this?
- What could have been improved in terms of evidence use in HPV/malaria vaccine policymaking?

Finally, would you recommend any additional individuals I should speak with? Additionally, would you be able to share any relevant documents that could provide further insights into our discussion and assist in constructing a timeline of key events?

**Thank you for your time. If you are comfortable with it, I may reach out for any necessary clarifications.**
