# Supplementary material for: Examining evidence use in vaccine policymaking in Kenya: a qualitative case study
Source: Health Policy Plan. 2026 Aug 10;41(9):1511–26. doi: 10.1093/heapol/czag100 (PMC13678818; doi:10.1093/heapol/czag100)
Supplement: Supplementary file 1 [file czag100_supplementary_data.zip › Supp file 9.docx]

**Supplementary file 9: Coding scheme linking study objectives, frameworks and codes**

| Study Objective | Guiding Framework | Main Coding Categories | Illustrative sub-codes |
| --- | --- | --- | --- |
| 1. To identify the types of evidence used in vaccine policymaking | **Spectrum approach** | **A. Types of Evidence** | - *Scientific evidence*: e.g., research studies, surveillance data, cost-effectiveness analyses. - *Tacit evidence*: e.g., expert opinions, program experience - *Evidence “in the middle”*: e.g., WHO position papers, national strategic plans and guidelines - *Source of evidence*: global, regional, or local. |
| 2. To examine how evidence and other factors interacted to influence policy | **Carol Weiss's models for research use** | **B. Models of Evidence Use** | - *Instrumental use*: evidence directly used to inform policy decisions. - *Political use*: evidence used to justify or legitimize choices. - *Interactive use*: evidence and other factors influenced decisions - *Imposed*: evidence use tied to donor or national government requirement |
